# Caseous calcification of the mitral annulus associated with a complete atrioventricular block: a case report

**DOI:** 10.1093/ehjcr/ytaf079

**Published:** 2025-02-14

**Authors:** Keisuke Kojima, Yasuhiro Oga, Junichiro Nishi

**Affiliations:** Department of Cardiology, Aso Iizuka Hospital, Yoshio-machi 3-83, Iizuka, Fukuoka-ken 8208505, Japan; Department of Cardiology, Aso Iizuka Hospital, Yoshio-machi 3-83, Iizuka, Fukuoka-ken 8208505, Japan; Department of Cardiology, Aso Iizuka Hospital, Yoshio-machi 3-83, Iizuka, Fukuoka-ken 8208505, Japan

**Keywords:** Caseous calcification of the mitral annulus, Complete atrioventricular block, Conduction disturbance, Cardiac mass, Case report

## Abstract

**Background:**

Caseous calcification of the mitral annulus (CCMA), a subtype of mitral annulus calcification, is rarely encountered. Although most cases of CCMA are asymptomatic and have a benign course, there are several reports of mitral valve dysfunction, stroke, and myocardial infarction. However, few reports have been published on conduction disturbances.

**Case summary:**

We encountered a case of an atrioventricular block, which is a rarely reported complication, in a 70-year-old woman who presented with heart failure. The patient’s anatomy suggested a conduction disturbance caused by CCMA extending from the posterior apex of the mitral annulus to the ventricular septum. Our heart team discussed whether surgical resection should be performed. We concluded that bradycardia was the most likely cause of the current symptoms and that resection of the extensively infiltrated calcification was risky; therefore, we decided to proceed with pacemaker implantation followed by careful observation of the mass. The patient had undergone permanent pacemaker implantation and has been asymptomatic ever since.

**Discussion:**

Few reports on conduction abnormalities caused by CCMA have been published. A mass involving the left ventricular septum and posterior mitral annulus may lead to conduction abnormalities, such as a complete atrioventricular block, in the future. We suggest that careful follow-up is required for CCMA, as it has been determined that surgical intervention is not required.

**Learning points**

- Although caseous calcification of the mitral annulus (CCMA) is a rare form of chronic degeneration, it may cause conduction abnormalities, particularly involving the left ventricular septum.
- Patients with CCMA should be followed up carefully for progression of conduction defects, even if they are asymptomatic.
- Caseous calcification of the mitral annulus may tend to gradually increase in size, necessitating close monitoring for progression of the valvular disease and symptoms. Surgical treatment should be considered in consultation with the heart team, factoring in the patient’s operative risk and clinical status.

## Introduction

Mitral annulus calcification (MAC) is defined as the chronic degeneration of the mitral valve fibrous ring. Mitral annulus calcification is a common finding in older adults, particularly in women and patients with end-stage renal disease undergoing dialysis. Caseous calcification of the mitral annulus (CCMA) is a highly luminous structure and is considered a rare subtype of MAC.^1^

Caseous calcification of the mitral annulus is very infrequent. The prevalence of CCMA was 0.63% in the MAC cases and 2.7% in the autopsy cases, respectively. Most of the patients are asymptomatic, and this is often an incidental finding in echocardiography.^1,2^

The CCMA is characterized by large, smooth margins and a rounded shape, in contrast to a typical MAC. It predominantly occurs in women and older adults and is consistently located at the posterior apex of the mitral annulus.^2,3^ Notably, the white ‘caseous’ structures are observed intraoperatively and at autopsy.^3,4^ However, the underlying mechanisms remain unclear.

Although there are several reports on mitral valve dysfunction (stenosis or regurgitation), stroke, and myocardial infarction,^5,6^ few reports have been published on conduction disturbances.

Here, we present a rare case of a patient with a large CCMA related to a complete atrioventricular block (CAVB).

## Summary figure

**Figure:**
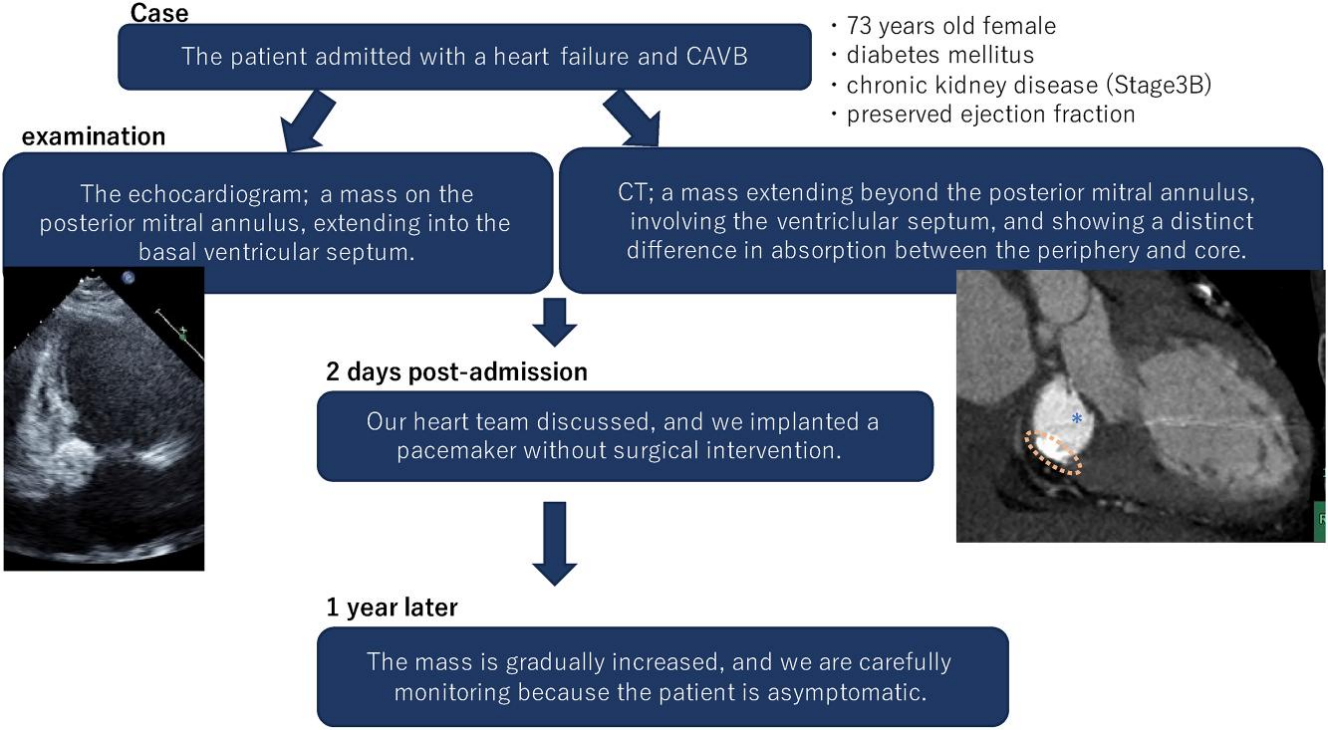


## Case presentation

A 73-year-old woman experienced shortness of breath on exertion for 2 months. The symptoms of shortness of breath gradually worsened, and dyspnoea of effort with light exertion severely interfered with her daily life. Her symptoms were classified as New York Heart Association (NYHA) Class Ⅲ. The patient visited a nearby clinic and was referred to our department with bradycardia (40 beats/min) and CAVB on admission.

**Figure 1:**
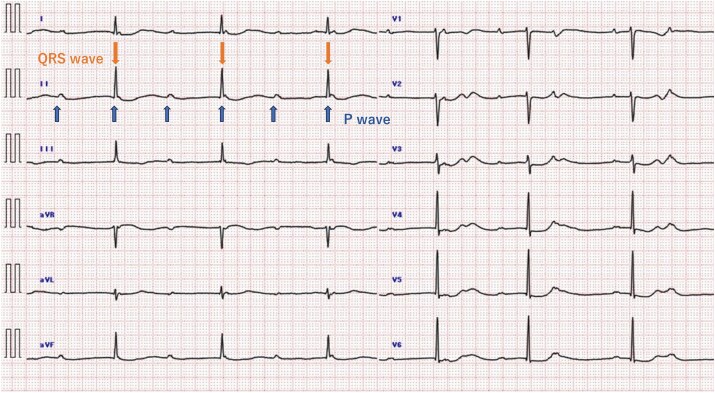
Electrocardiogram at presentation: complete atrioventricular block with junctional escape rhythm. A rate of 40 beats/min (down arrows) and a P wave rate of 70 beats/min (up arrows).

The patient took oral medications for diabetes mellitus, hypertension, and dyslipidaemia. Additionally, chronic kidney disease (CKD) had been diagnosed, with an estimated glomerular filtration rate of 28 mL/min/1.73 m^2^, classifying it as Stage 3b CKD. No history of smoking or alcohol consumption was reported. Upon visiting our hospital, an electrocardiogram showed a CAVB with an escape rhythm from the atrioventricular junction (ventricular rate of 40 beats/min) (*Figure 1*). The patient had pulmonary congestion, the jugular vein dilatation, bilateral pitting oedema, and an elevated B-type natriuretic peptide level of 544 pg/mL (reference: <18.4 pg/mL). The serum troponin levels were not elevated.

A cyst was found in the thyroid gland, but there was no evidence of abnormal function [thyroid stimulating hormone, 1.75 μIU/mL (reference: 0.61–4.23 μIU/mL); free thyroxine, 1.19 ng/dL (reference: 0.7–1.48 ng/dL)]. No abnormalities in electrolyte levels were observed.

**Figure 2:**
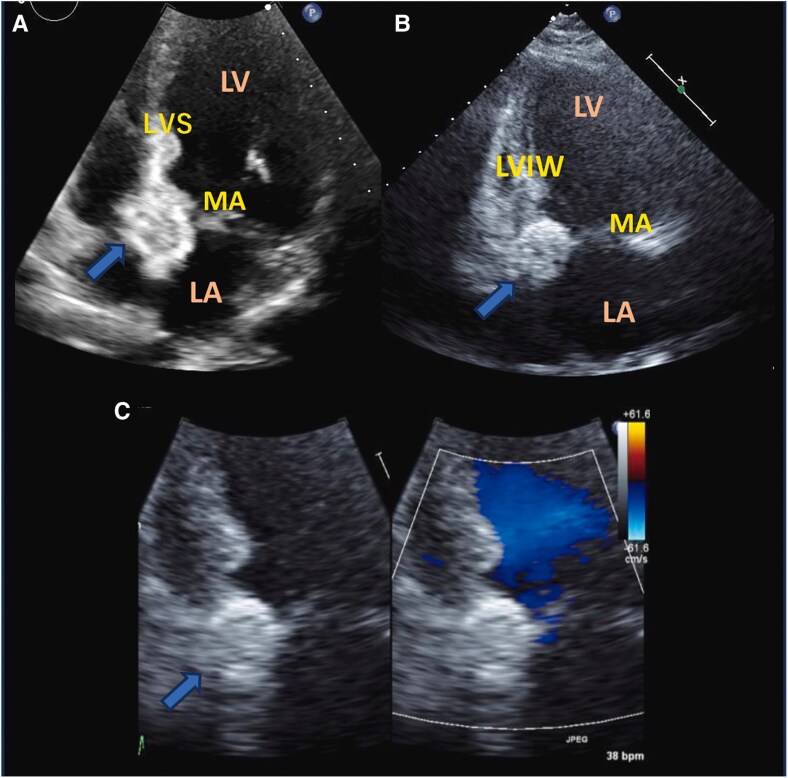
Transthoracic echocardiogram at presentation: the apical four-chamber view (*A*) and the apical two-chamber view (*B*). The mass has a high-intensity area of margins and an echolucent area of intensity, and there is no colour signal (*C*). LA, left atrium; LV, left ventricle; MA, mitral annulus; LVS, left ventricular septum; LVIW, left ventricle inferior wall.

**Figure 3:**
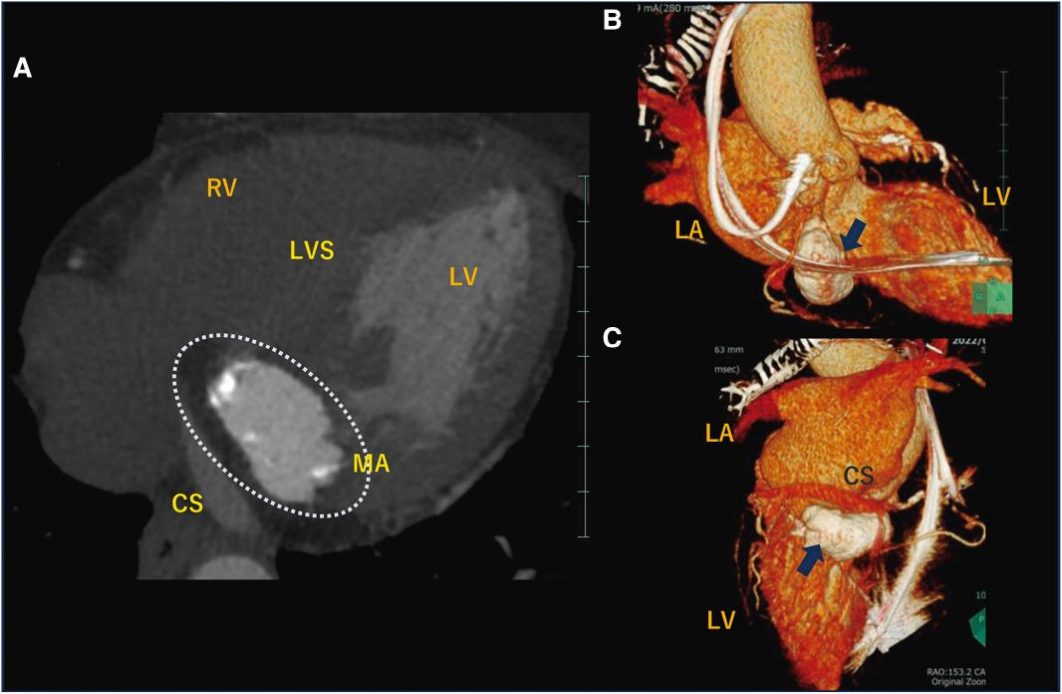
Computed tomography with contrast: the massive calcification of the posterior mitral leaflet (*A*). Three-dimensional volume rendering showing the mass from the right oblique view (*B*) and the posterior view (*C*) (removing the right atrium and ventricle). LA, left atrium; LV, left ventricle; RV, right ventricle; MA, mitral annulus; CS, coronary sinus; LVS, left ventricular septum.

**Figure 4:**
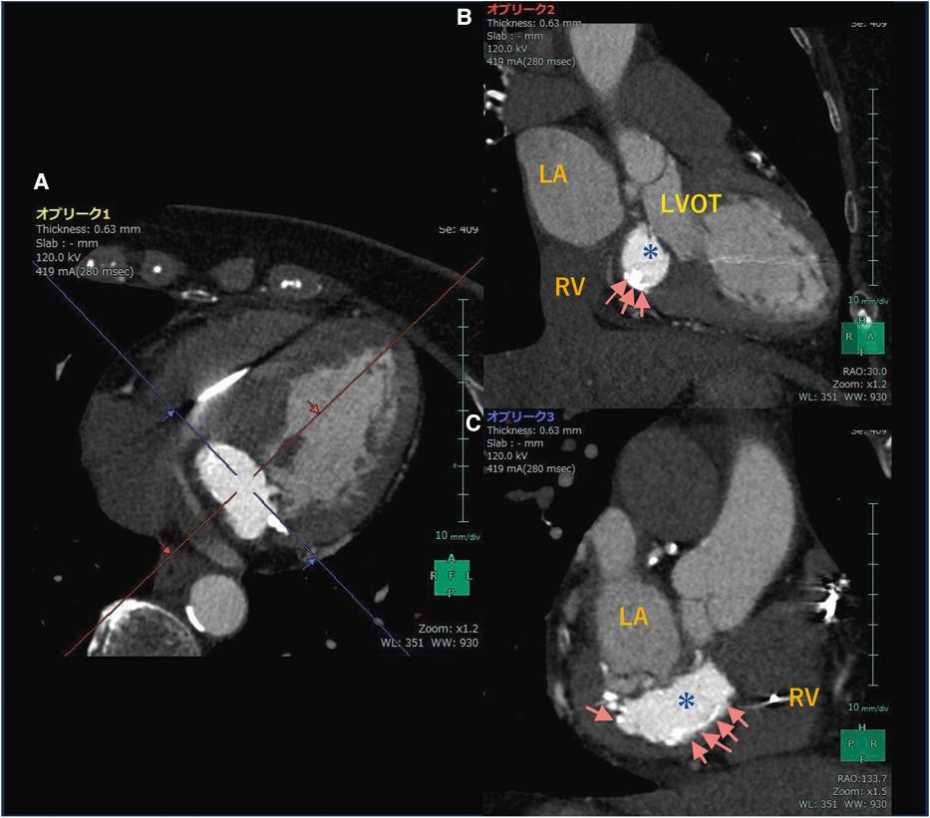
Computed tomography with contrast: the short-axial view (*A*). The right oblique view is shown with reconstruction along red arrows (*B*). The posterior view is shown with reconstruction along blue arrows (*C*). Changes in window level (250 → 500) and width (800 → 1600) show differences in the absorption area between the edges and contents of the mass. The content has relatively lower absorption than the limbus, indicating a mixture of calcified components (arrows) and liquid components (asterisk mark). LA, left atrium; RV, right ventricle; LVOT, left ventricle outflow tract.

Transthoracic echocardiography showed a normal ejection fraction of 60% and incidentally revealed a mass without an acoustic shadow on the posterior mitral annulus, extending into the basal ventricular septum. The periphery of the mass was highly luminous, indicating calcification, while the core appeared echolucent, suggesting CCMA. The mass presented as a dense, echogenic lesion measuring 17 × 19 mm, fixed posteriorly with immobile borders (Supplementary material online and *Figure 2*). Mild mitral findings were noted, and mitral stenosis was absent. Computed tomography (CT) revealed a mass extending beyond the posterior mitral annulus, and showing a distinct difference in absorption between the periphery and core. The periphery exhibited high density, whereas the core displayed relatively low absorption. The mass was sizable, seemingly involving the basal septum of the left ventricle (*Figures 3* and *4*), a region encompassing the membranous septum associated with atrioventricular conduction. Specialized cardiac muscles with conducting systems are considered intrinsic to this area. The CT scan revealed no significant stenosis within the coronary arteries.

**Figure 5:**
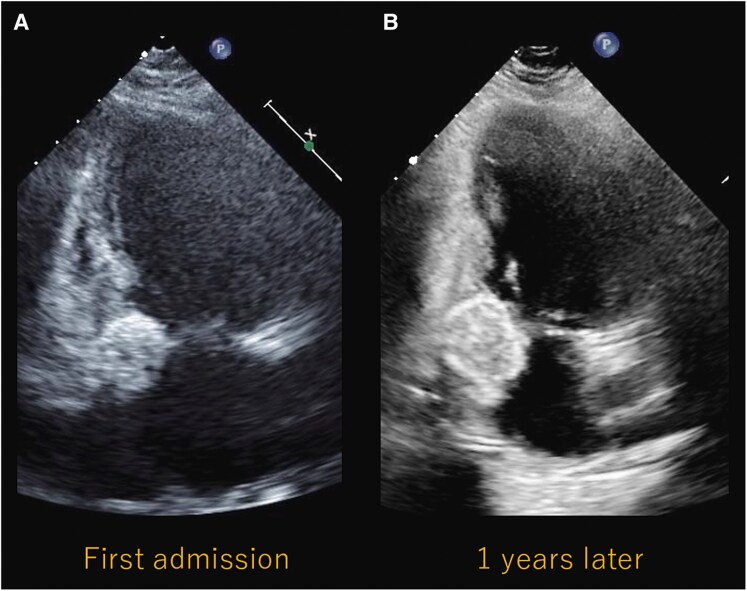
Transthoracic echocardiogram at presentation (*A*) and 1 year later (*B*). These are from the apical two-chamber view. The mass had increased in size from 17 × 19 mm to 23 × 26 mm.

We discussed how to formulate a treatment plan with the heart team, including a cardiovascular surgeon. The calcified area extended beyond the annulus, involving a significant portion of the myocardium. Given the high surgical risk associated with removing the extensive calcification and the likelihood of addressing the conduction disturbance with pacemaker implantation, we opted for a conservative approach. The patient started taking diuretics (furosemide 20 mg per day), and a DDD pacemaker (Evity 8 DR-T ProMRI, BIOTRONIC) implantation was performed using standard procedures 2 days post-admission. Following the procedure and with amelioration of bradycardia, the symptoms resolved, and the patient has remained disability-free to date (NYHA Class I). The symptom of shortness of breath was thought to be mainly due to bradycardia. Given the absence of symptoms to date and the lack of valvular disease or cerebral infarction, we opted for follow-up with meticulous echocardiography. One year post-initial admission, the volume of the calcification has gradually increased (*Figure 5*), from 17 × 19 mm to 23 × 26 mm in the apical two-chamber view. However, it has not contributed to the worsening of the valvular disease, and the patient is able to lead a symptom-free daily life. At present, careful follow-up is being carried out without treatment.

## Discussion

Caseous calcification of the mitral annulus, a dystrophic calcification derived from degenerative myocardial disease, is a rare variant of MAC.^1^ Transthoracic echocardiography is visually characterized by edge hyperintensity without an acoustic shadow (reflecting calcification) despite internal echo-lucency (reflecting soft tissue with necrotic debris). In most cases, other lesions like cardiac tumours and myocardial abscesses can be differentiated using imaging because there is no calcification, and blood flow is often present.^5^

Computed tomography is also an effective tool for diagnosing CCMA. On CT, CCMA is characterized by a high-density, ring-like mass featuring a central area with no contrast enhancement.^7^

The specific mechanism underlying the liquefaction of the CCMA remains unclear. Mitral annulus calcification is believed to arise from disrupted calcium-phosphate metabolism. Recurrent mechanical forces and endothelial damage lead to the deposition of calcium salts in the myocardial tissue exposed, notably in the aortic and mitral valve tissues. A comparable mechanism has been suggested for the CCMA.^8^ Various reports describe pathological characteristics, including amorphous, eosinophilic, and acellular material encircled by macrophages and lymphocytes, accompanied by calcification and necrosis.^4,9^

Mitral annulus calcification is linked to conduction system disease^1^; thus, CCMA may pose a risk of conduction disturbance. The spread of calcific deposits to the atrioventricular node and bundle of His areas might explain conduction disease. Few reports detail arrhythmias like atrioventricular block in MAC patients, and even fewer in CCMA patients.^10^ In the present case, the left ventricular region extended into the muscular septum, affecting a specialized conduction system. Patients with CCMA should undergo regular electrocardiograms and close monitoring for conduction disturbances.

Caseous calcification of the mitral annulus is a degenerative change that typically increases in size over time. However, in some cases, it may show partial shrinkage upon observation without surgery, indicating dynamic progression.^11^ There is no consensus regarding CCMA management, particularly on whether surgical removal of the lesion is warranted. The current ACC/AHA guidelines recommend that intervention for calcific severe mitral stenosis should be considered only in patients with severe symptoms due to the high operative risk associated with the procedure.^12^ Other reports suggest avoiding early surgical interventions, as it typically represents a benign process, not proliferative tumour cell changes.^5^ In the present case, given that the arrhythmia was expected to resolve with pacemaker implantation and considering high surgical risks, our heart team opted for a conservative treatment approach.

## Conclusion

Caseous calcification of the mitral annulus is a rare degenerative change that may result in conduction disturbances, such as atrioventricular block. Patients diagnosed with CCMA extending to the left ventricular septum should be carefully monitored, as the condition can often be managed with pacemaker implantation.

## Supplementary Material

ytaf079_Supplementary_Data

## Data Availability

The data underlying this article will be shared upon reasonable request to the corresponding author.

## Lead author biography

Dr Keisuke Kojima was born in Fukuoka, Japan in 1991. He graduated from Kyushu University in 2016. He experienced this case at the Aso Iizuka Hospital in 2022. His main interests are interventional cardiology and echocardiography.

## Notes

**Consent:** Written informed consent was obtained from the patient for the publication of this case report and the accompanying images in line with COPE guidance. A copy of the written consent form is available for review by the journal editor.

**Funding:** None declared.
